# Increased RV:LV ratio on chest CT-angiogram in COVID-19 is a marker of adverse outcomes

**DOI:** 10.1186/s43044-022-00274-w

**Published:** 2022-05-08

**Authors:** Ran Tao, Zuzana Burivalova, S. Carolina Masri, Naga Dharmavaram, Aurangzeb Baber, Roderick Deaño, Timothy Hess, Ravi Dhingra, James Runo, Nizar Jarjour, Rebecca R. Vanderpool, Naomi Chesler, Joanna E. Kusmirek, Marlowe Eldridge, Christopher Francois, Farhan Raza

**Affiliations:** 1 Department of Medicine, CSC-E5/582B, University of Wisconsin Hospitals and Clinics, University of Wisconsin-Madison, 600 Highland Avenue, Madison, WI 53792 USA; 2 Nelson Institute for Environmental Studies, University of Wisconsin-Madison, 600 Highland Avenue, Madison, WI 53792 USA; 3 Department of Medicine-Division of Cardiology, University of Wisconsin-Madison, 600 Highland Avenue, Madison, WI 53792 USA; 4 Department of Medicine-Division of Pulmonary and Critical Care, University of Wisconsin-Madison, 600 Highland Avenue, Madison, WI 53792 USA; 5 Department of Biomedical Engineering, The University of Arizona, 1127 E. James E. Rogers Way, Tucson, AZ 85721 USA; 6 Department of Biomedical Engineering, The Henry Samueli School of Engineering, University of California, Irvine, Irvine, CA 92697 USA; 7 Department of Radiology, University of Wisconsin-Madison, 600 Highland Avenue, Madison, WI 53792 USA; 8 Department of Pediatrics, University of Wisconsin-Madison, 600 Highland Avenue, Madison, WI 53792 USA; 9 Mayo Clinic Radiology, 200 First St. SW, Rochester, MN 55905 USA

**Keywords:** COVID-19, Right ventricular dilation, RV:LV ratio, Chest CT-angiogram

## Abstract

**Background:**

Right ventricular (RV) dilation has been used to predict adverse outcomes in acute pulmonary conditions. It has been used to categorize the severity of novel coronavirus infection (COVID-19) infection. Our study aimed to use chest CT-angiogram (CTA) to assess if increased RV dilation, quantified as an increased RV:LV (left ventricle) ratio, is associated with adverse outcomes in the COVID-19 infection, and if it occurs out of proportion to lung parenchymal disease.

**Results:**

We reviewed clinical, laboratory, and chest CTA findings in COVID-19 patients (*n* = 100), and two control groups: normal subjects (*n* = 10) and subjects with organizing pneumonia (*n* = 10). On a chest CTA, we measured basal dimensions of the RV and LV in a focused 4-chamber view, and dimensions of pulmonary artery (PA) and aorta (AO) at the PA bifurcation level. Among the COVID-19 cohort, a higher RV:LV ratio was correlated with adverse outcomes, defined as ICU admission, intubation, or death. In patients with adverse outcomes, the RV:LV ratio was 1.06 ± 0.10, versus 0.95 ± 0.15 in patients without adverse outcomes. Among the adverse outcomes group, compared to the control subjects with organizing pneumonia, the lung parenchymal damage was lower (22.6 ± 9.0 vs. 32.7 ± 6.6), yet the RV:LV ratio was higher (1.06 ± 0.14 vs. 0.89 ± 0.07). In ROC analysis, RV:LV ratio had an AUC = 0.707 with an optimal cutoff of RV:LV ≥ 1.1 as a predictor of adverse outcomes. In a validation cohort (*n* = 25), an RV:LV ≥ 1.1 as a cutoff predicted adverse outcomes with an odds ratio of 76:1.

**Conclusions:**

In COVID-19 patients, RV:LV ratio ≥ 1.1 on CTA chest is correlated with adverse outcomes. RV dilation in COVID-19 is out of proportion to parenchymal lung damage, pointing toward a vascular and/or thrombotic injury in the lungs.

## Background

Most Coronavirus Disease 2019 (COVID-19) infections are asymptomatic or mild, yet severe infections can occur and lead to multiorgan failure and death [1–6]. Patients who are older or have pre-existing conditions are at a higher risk of developing severe symptoms [2, 4, 5, 7, 8]. Predictors of severe disease include lymphopenia, elevated levels of d-dimer, lactate dehydrogenase, ferritin, troponin, and brain natriuretic peptide (BNP) [3, 4, 9]. Studies using computerized tomography (CT) of the chest found an association of the severity of COVID-19 with extensive consolidation and multiple lung segment involvement [10–13]. However, the use of CT in routine diagnosis has been discouraged due to a lack of specificity and the potential of exposing the imaging suite and personnel to the disease [14, 15].

Multiple groups have proposed the pathogenesis of circulatory dysfunction in COVID-19. COVID-19 infection may induce direct myocardial injury (myocarditis), vascular injury in the pulmonary circulation, hypercoagulable state, and stress-induced cardiomyopathy, resulting in right-sided heart failure [7, 16–18]. While most studies of right heart failure are echocardiogram-based [6, 19, 20], prior chest CT studies had reported that an increased right ventricle (RV) to left ventricle (LV) ratio predicts adverse outcomes in acute pulmonary conditions, such as acute respiratory distress syndrome (ARDS) and acute pulmonary embolism (PE) [21–25]. The aim of the current study is to determine if an increased RV:LV ratio on a chest CT-angiogram (CTA) in COVID-19 patients is associated with adverse clinical outcomes, and if an increased RV:LV ratio occurs out of proportion to lung parenchymal disease.

## Methods

### Study population

Between March 1 and September 30, 2020, 4543 patients were tested positive for COVID-19 infection in the University of Wisconsin health system. Among the positive cases, 100 consecutive patients were included in this study, who underwent chest CTA chest due to an elevated d-dimer level and suspected acute PE within 35 days of both tests. Subjects who had a COVID+ test and CTA chest more than 35 days apart were excluded from the study. We quantified the baseline characteristics, clinical course, and CTA chest findings of COVID-19 patients and the control groups. We classified the COVID-19 cohort based on adverse outcomes (defined as ICU admission, intubation, or death), whereby COVID-AO+ signifies the subgroup adverse outcomes and COVID-AO− signifies the subgroup with no adverse outcomes.

As a control group, we identified two cohorts of patients who underwent CTA chest for suspected acute PE or hypoxemia; but did not have a PE and did not have COVID-19 infection. The first control group included ten patients with suspected acute PE from January to April 2020, but had neither PE nor COVID-19 *(Normal control)*. The second control group included ten patients with organizing pneumonia *(Org-PNA control)* between 2012–2019. Both control groups were selected to match with the COVID-19 group for age and pre-existing conditions. The organizing pneumonia group was chosen to differentiate the impact of chronic lung parenchymal disease from the COVID-19 infection.

Moreover, we selected a separate validation cohort of 25 consecutive patients with COVID-19, who underwent CTA chest between October 1 and October 30, 2020, for suspected PE (but did not have a PE).

### CTA chest acquisition

According to clinical standard protocols for pulmonary embolism, all patients were scanned without ECG gating on 64-slice and 256-slice scanners. Imaging was performed after intravenous contrast administration, and contrast enhancement was present in the pulmonary and systemic circulation.

### Chamber quantification on CT images

**Fig. 1:**
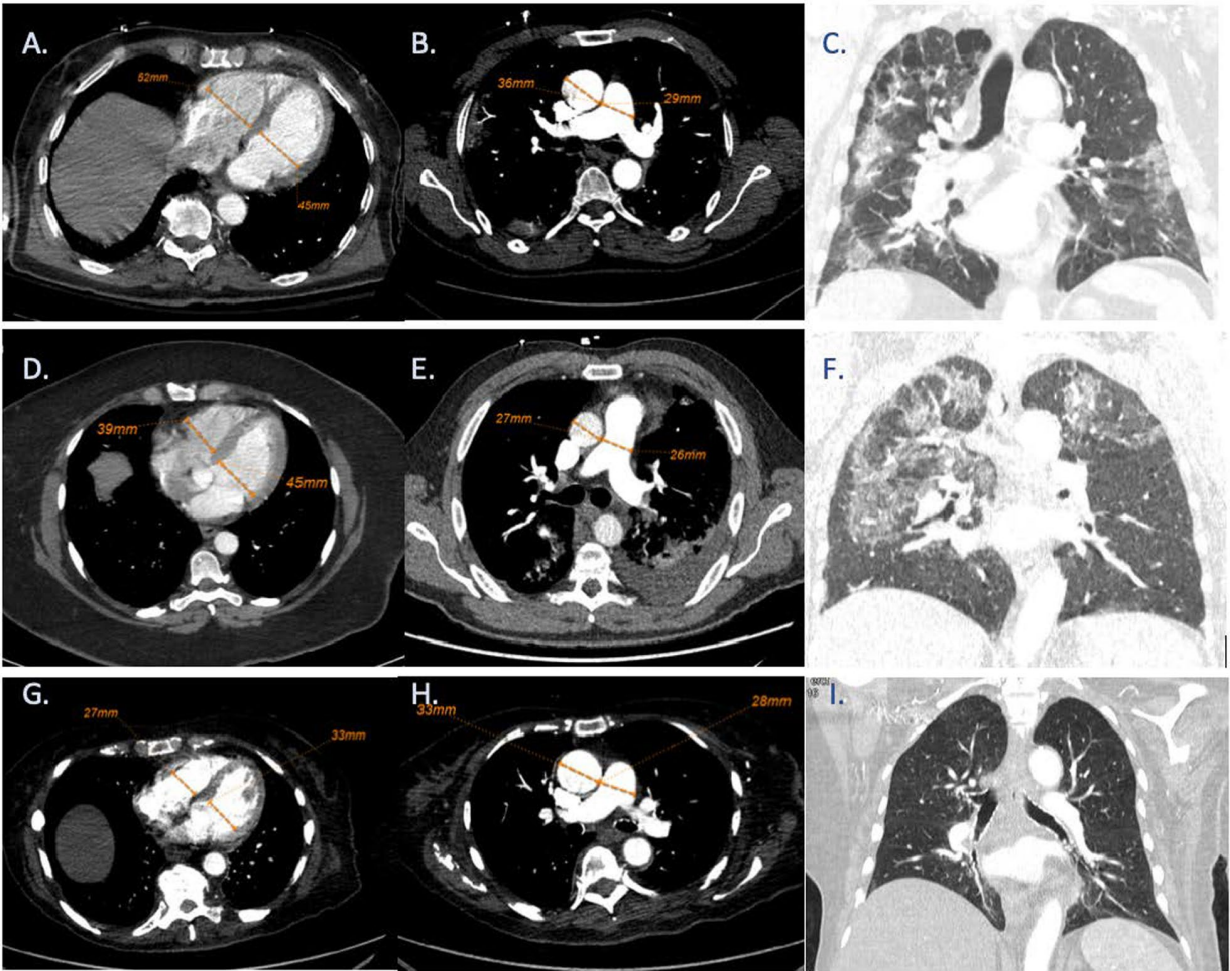
Representative images of quantitative measurements and lung parenchyma in COVID-19, Org-PNA, and normal control cohort. **A**–**C** COVID-19 (RV 52 mm, LV 45 mm, RV:LV 1.15, PA 29 mm, AO 36 mm, PA:AO 0.8). **D**–**F** Org-PNA (RV 39 mm, LV 45 mm, RV:LV 0.86, PA 26 mm, AO 27 mm, PA:AO 0.96). **G**–**I** Normal (RV 27 mm, LV 33 mm, RV:LV 0.81, PA 28 mm, AO 33 mm, PA:AO 0.84). *COVID-19* novel coronavirus, *RV* right ventricle, *LV* left ventricle, *AO* aorta, *PA* pulmonary artery, *Org-PNA* organizing pneumonia

The CT scans were independently and retrospectively reviewed by an experienced cardiothoracic radiologist (CF, 13 years' experience) and a cardiac imaging specialist (FR). All chamber quantification measurements were performed as represented in Fig. 1. The basal diameters of the RV and LV (maximum distance between the ventricular endocardium and the interventricular septum) were measured in 4-chamber, axial, and short-axis views. The 4-chamber views were used for analysis. At the level of the pulmonary artery (PA) bifurcation, the inner wall-to-inner wall diameters of the aorta (AO) and the PA were obtained in the axial plane (PA measured just proximal to the bifurcation).

### Chest CT COVID-19 severity score

**Fig. 2:**
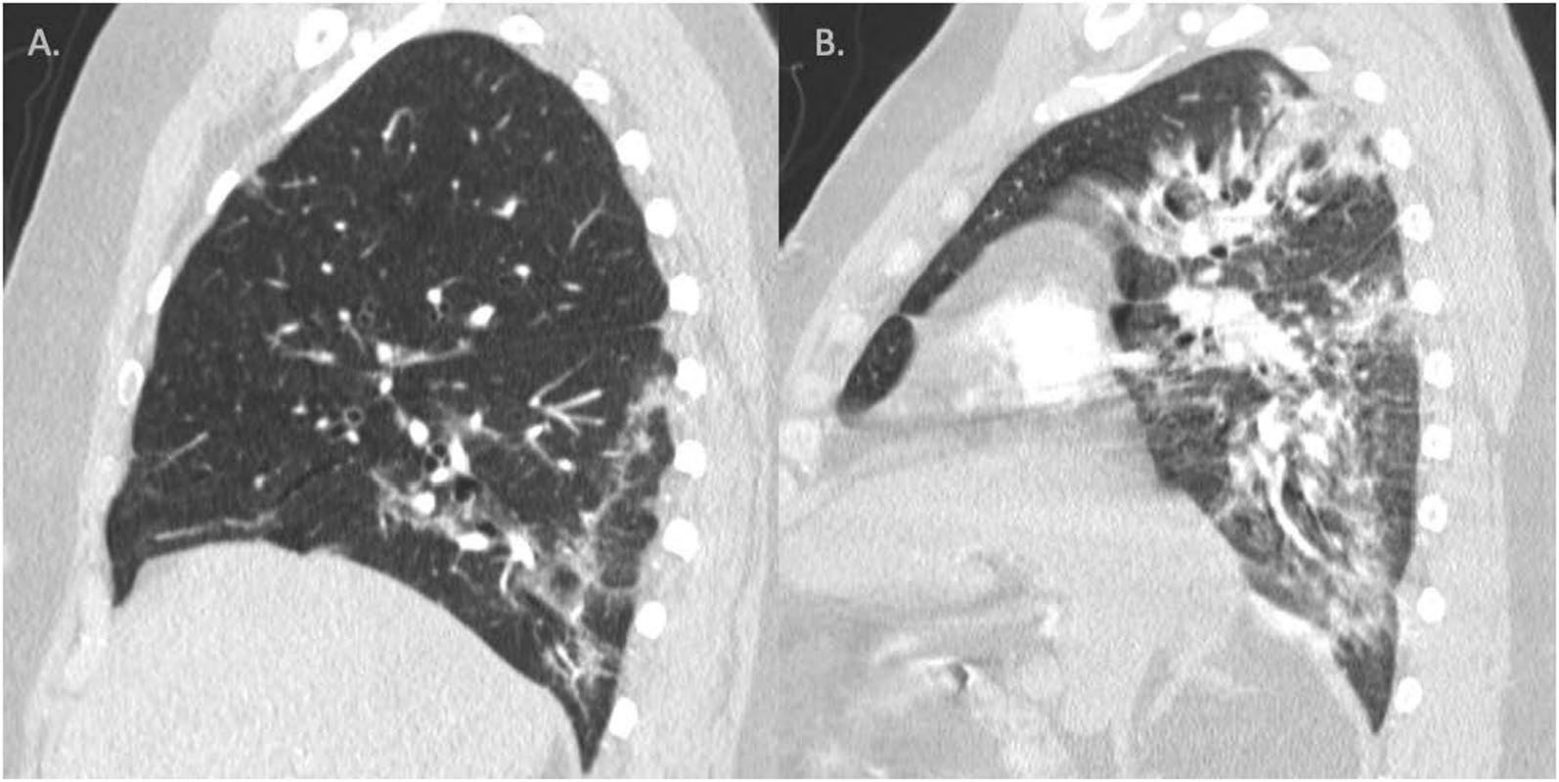
Representative images demonstrating the difference between a CT-SS scoring system. **A** Severity score = 1, **B** severity score = 2. *CT-SS* CT scoring system [26]

To report the severity of parenchymal lung involvement in COVID-19, we utilized the scoring system proposed by Yang et al. (CT-SS: CT scoring system) [26]. As recommended by Yang et al., we reported the lung opacities in all twenty lung regions on chest CT based on the degree of parenchymal opacification percentage (Fig. 2). For each lung region, 0% opacification = score 0, 0–50% opacification = score 1, and > 50% opacification = score 2. The CT-SS was defined as the sum of the individual scored in the 20 lung segment regions, i.e., ranging from 0 to 40 points. All CT images were reviewed by Drs. Francois and Raza, blinded to the clinical data and laboratory indicators.

### Statistical analyses

Continuous variables were presented as mean ± standard deviation (SD), or range when appropriate, and their difference between groups was assessed using analysis of variance (ANOVA), where data were normally distributed. Categorical variables were analyzed using Fisher's exact test. For RV:LV analysis, we assessed the inter- and intra-observer bias in CT measurements using the Bland–Altman (or Tukey's Mean Difference) plot, as recommended by Gow et al. 2008 [27]. To test the difference in RV:LV and PA:AO ratio between groups, we carried out a post-hoc pairwise comparison, adjusted for multiple testing with Bonferroni correction. Receiver operating curve (ROC) analysis was performed to detect area under the curve (AUC) to determine the discriminatory ability of the RV:LV ratio to predict adverse outcomes in COVID-19 patients. To test for potential confounding factors, we fit a simple linear regression to the RV:LV ratio and BMI and age, as well as PA:AO ratio and BMI and age. We also tested for a correlation between the RV:LV ratio and the CT-SS. All analyses were performed in SPSS v22.0 (IBM Corp. Armonk, NY, USA) and R (version 4.0.0) [28].

## Results

### The COVID-19 cohort

The baseline clinical features and significant laboratory findings at the time of admission of the 100 patients with COVID-19 infection are included in Table 1. The mean age was 55.1 ± 14.9 years, and 55 were female. The presenting symptoms included: dyspnea (81%), cough (80%), and fever (61%). Fifty-eight patients had at least one pre-existing condition or were active smokers. Among the 100 patients, 57 were hospitalized for management. Six patients received chloroquine, five patients received convalescent plasma, eight patients received Remdesivir, and 21 patients received both Remdesivir and convalescent plasma. Twenty-three patients received therapeutic anticoagulation, whereas the rest received prophylactic anticoagulation. The timeline between positive COVID test and CTA chest acquisition was 10.3 ± 12.5 days (with a range from 0 to 34 days).

**Table 1:** Clinical features of COVID-19 patients in the study and validation cohorts

Parameters	COVID-19 study cohort (*n* = 100)	COVID-19 validation cohort (*n* = 25)	*p* value
Age (years)	55.1 ± 14.9	63.4 ± 12.9	< 0.01
Female, *n* (%)	55 (55%)	8 (32%)	0.039
Symptoms, *n* (%)			
– Shortness of breath	81 (81%)	18 (72%)	0.32
– Cough	80 (80%)	17 (68%)	0.20
– Fever	61 (61%)	14 (56%)	0.60
– Sore-throat	16 (16%)	3 (12%)	0.62
Past medical history, *n* (%)			
– Asthma	21 (21%)	5 (20%)	0.91
– OSA	16 (16%)	8 (32%)	0.07
– HTN	46 (46%)	13 (52%)	0.59
– DM	30 (30%)	9 (36%)	0.56
– CAD	14 (14%)	5 (20%)	0.45
Symptom-onset → COVID-19+ (days)	5.3 ± 6.3	3.2 ± 2.1	< 0.01
COVID-19+ → chest CTA time (days)	10.3 ± 12.5	3.3 ± 3.7	< 0.01
Outcomes, *n* (%)			
– Inpatient	58 (58%)	14 (56%)	0.85
– Length of hospital stay (days)	11.1 ± 9.1	10.1 ± 12.7	0.78
– Supplemental oxygen	46 (46%)	11 (44%)	0.86
– ICU admission	24 (24%)	3 (12%)	0.19
– Intubation	19 (19%)	3 (12%)	0.41
– Death	10 (10%)	2 (7%)	0.76
COVID medication, *n* (%)			
– Chloroquine	6 (6%)	0 (0%)	0.21
– Convalescent plasma	5 (5%)	2 (8%)	0.13
– Remdesivir	8 (8%)	0 (0%)	0.14
– Remdesivir + convalescent plasma	21 (21%)	9 (36%)	0.11
Laboratory data			
D-dimer (mcg/mL)	1.7 ± 2.9	1.1 ± 0.6	0.05
CRP (mg/dL)	9.1 ± 8.6	14.5 ± 9.6	0.08
Lactate (mmol/L)	2.0 ± 1.2	1.9 ± 1.3	0.84
Absolute lymphocytes (/mcL)	1470 ± 1100	1090 ± 650	0.03
Platelets (K/micro-L)	221 ± 77	201 ± 70	0.22
Creatinine (mg/dL)	1.1 ± 1.8	1.1 ± 0.3	0.83

Values are mean ± SD

*COVID-19* novel coronavirus, *OSA* obstructive sleep apnea, *HTN* hypertension, *DM* diabetes, *CAD* coronary artery disease, *ICU* intensive care unit, *CRP* C-reactive protein

### The RV:LV ratio is elevated in patients with COVID-19

Across all COVID-19 positive patients, the average LV and RV basal diameters were 39.7 ± 7.0 mm and 38.5 ± 6.4 mm, respectively. The average RV:LV ratio was 0.98 ± 0.15. Compared with the two control groups, the average RV:LV ratio was higher in COVID-positive patients than in the normal control (*p* < 0.001) and than in the patients with organizing pneumonia (*p* < 0.01).

On inter- and intra-observer variability analysis, we found an acceptable level of variation in terms of the RV and LV measurements on CTA chest (*r* = 0.922 for inter-observer variability 0.915 for intra-observer variability). Neither inter- nor intra-observer variability changed with the variable mean. Moreover, among the 100 patients, only 11 patients underwent transthoracic echocardiogram (TTE). The timeline between the CTA chest and TTE was 1.4 ± 1.1 days. The RV:LV ratio was similar between CTA-based vs. TTE-based methods (1.06 ± 0.15 vs. 1.06 ± 0.15, *p* = 0.43).

### COVID-AO+ versus COVID-AO−

Among the overall COVID cohort (*n* = 100), 25 patients had an adverse outcome (COVID-AO+), defined as ICU admission (24 events), endotracheal intubation (19 events), or death (10 events). The remaining 75 patients had no adverse outcomes (COVID-AO−). The baseline characteristics of the two groups are compared in Table 2. The COVID-AO+ had a longer hospital stay (17 ± 11 vs. 7 ± 3 days in COVID-AO−) and a higher rate of supplemental oxygen requirement. COVID-AO+ group also had more abnormal laboratory markers at the time of COVID-19 diagnosis (Table 2).

**Table 2:** Comparison of COVID-19 cohorts (COVID-AO+ and COVID-AO−) and the control cohorts (normal and Org-PNA)

	COVID-AO− (*n* = 75)	COVID-AO+ (*n* = 25)	Normal control (*n* = 10)	Org-PNA control (*n* = 10)	*p* value
Age	53 ± 14	60 ± 16	64 ± 6.2	70 ± 16	0.001
Female, *n* (%)	43 (57%)	12 (48%)	3 (30%)	6 (60%)	n.s.
PMH, *n* (%)					
≥ 2 pre-existing conditions*	39 (52%)	15 (60%)	8 (80%)	9 (90%)	n.s.
Outcomes, *n* (%)					
– Inpatient	32 (43%)	25 (100%)			< 0.001
– LOS (days)	6.5 ± 3.5	16.9 ± 10.6			< 0.001
– Supplemental O_2_	21 (28%)	25 (100%)			< 0.001
Laboratory data					
D-dimer (mcg/mL)	1.5 ± 2.7	2.4 ± 3.4			0.26
CRP (mg/dL)	6.4 ± 5.9	14.6 ± 10.7			0.003
Lactate (mmol/L)	1.8 ± 0.6	2.2 ± 1.9			0.36
Absolute lymphocytes (/mcL)	1680 ± 1120	1110 ± 990			0.07
Platelets (K/micro-L)	225 ± 75	210 ± 84			0.43
Creatinine (mg/dL)	1.2 ± 2.1	1.0 ± 0.5			0.42
Chest CT findings					
RV basal diameter (mm)	38.3 ± 6.5	39.2 ± 5.9	33.7 ± 4.4	35.9 ± 2.8	n.s.
LV basal diameter (mm)	40.5 ± 6.9	37.3 ± 6.7	39.7 ± 2.5	40.4 ± 5.0	n.s.
RV:LV ratio	0.95 ± 0.15	1.06 ± 0.14	0.85 ± 0.08	0.89 ± 0.07	< 0.001
PA diameter (mm)	27.9 ± 4.7	26.8 ± 4.2	27.1 ± 3.4	30.5 ± 2.8	n.s.
AO diameter (mm)	30.6 ± 4.2	30.5 ± 3.8	32.5 ± 2.4	33.1 ± 3.7	n.s.
PA:AO	0.91 ± 0.13	0.88 ± 0.09	0.85 ± 0.10	0.93 ± 0.08	n.s.
CT severity score	13.8 ± 10.0	22.6 ± 9.0	0.0	32.7 ± 6.6	< 0.001^†^

Values are mean ± SD

*COVID-19* novel coronavirus, *COVID-AO*+ COVID-19 subgroup with adverse effects (defined as ICU admission, endotracheal intubation, or death), *COVID-AO− *COVID-19 subgroup without adverse effects, *Org-PNA* organizing pneumonia, *LOS* length of hospital stay, *CRP* C-reactive protein, *RV* right ventricle, *LV* left ventricle, *PA* pulmonary artery, *AO* aorta

*Pre-existing conditions included were lung disease (asthma/chronic obstructive lung disease/interstitial lung disease), obstructive sleep apnea, hypertension, diabetes, coronary artery disease, heart failure

^†^*p* value: excluding the normal control group

**Fig. 3:**
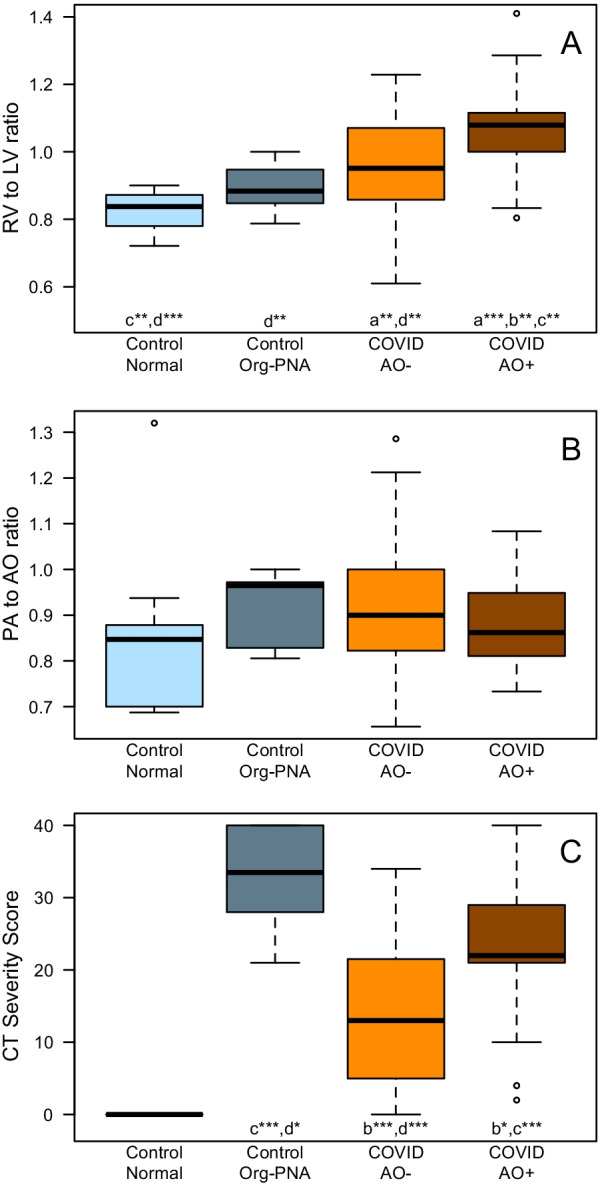
Comparison of RV:LV ratio, PA:AO ratio, and CT severity score between control and COVID-19 groups. Box and Whisker plots comparing **A** the ratio of RV to LV dimensions, **B** the ratio of PA to AO dimensions, and **C** the CT severity score (CT-SS). Horizontal lines represent median, boxes first and third quartile, whiskers 95%. Groups: control normal (control group without pneumonia); control Org-PNA (control group with organizing pneumonia); COVID-AO− (COVID-19 without adverse outcomes); COVID-AO+ (COVID-19 with adverse outcomes: death, or intubation, or ICU admission). Results of post-hoc pairwise comparison shown by letters (a, b, c, d for Control Normal, Control Org-PNA, COVID-AO−, COVID-AO+, respectively); significance level (adjusted for multiple testing using Bonferroni correction): *p < 0.05, **p < 0.01, ***p < 0.001. *RV* right ventricle, *LV* left ventricle, *AO* aorta, *PA* pulmonary artery, *COVID-19* novel coronavirus, *Org-PNA* organizing pneumonia

The COVID-AO+ subgroup had a higher RV:LV ratio (1.06 ± 0.14) than the COVID-AO− subgroup (0.95 ± 0.15, *p* < 0.01). When compared with the two control groups, the RV:LV ratio increased incrementally in the order of "normal cohort < Org-PNA cohort < COVID-AO− < COVID-AO+" (Fig. 3, Table 2)*.* The PA:AO ratio was similar between groups (Fig. 3, Table 2).

### The RV:LV ratio as a marker of adverse outcomes in COVID-19

**Fig. 4:**
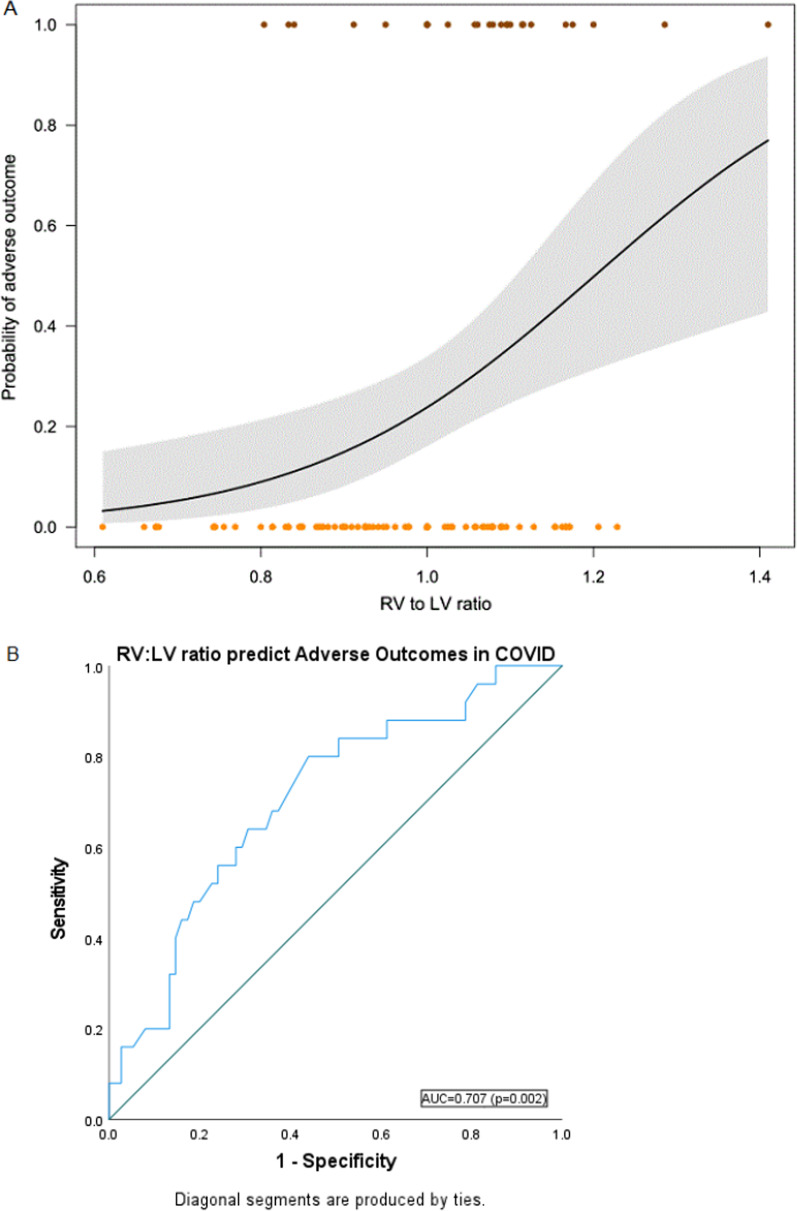
Correlation of RV:LV ratio with the probability of adverse outcomes (death, intubation, ICU admission) in COVID-19 cohort. **A** logistic regression (dots represent data: BROWN dots: adverse outcomes, ORANGE dots: no adverse outcomes; RV:LV ratio on the *x*-axis; black line represents the fit of the logistic regression, gray polygon represents 95% confidence interval). **B** Receiver operator curve of RV:LV ratio predicting adverse outcomes (AUC = 0.707, *p* = 0.002, CI 0.59–0.82, Optimal cutoff = 1.1)**.** *RV* right ventricle, *LV* left ventricle, *COVID-19* novel coronavirus

The probability of an adverse outcome increased with an increasing RV:LV ratio (Fig. 4A). We did not find evidence that this difference was explained by the potential confounding variables—neither BMI nor age were correlated with the RV:LV ratio. Receiver operating curve analysis of the RV:LV ratio predicting adverse outcomes revealed AUC = 0.707 (CI: 0.592–0.823, *p* = 0.002) with an optimal cutoff of 1.1. Among subgroups of COVID-19 patients with RV:LV ratio ≥ 1.1, 9/20 (45%) had adverse outcomes; and among COVID-19 patients with RV:LV ratio < 1.1, 16/80 (20%) had adverse outcomes.

### RV:LV ratio is higher and out of proportion to lung parenchymal damage in COVID-19

The parenchymal lung involvement was assessed using CT scoring system (CT-SS, Fig. 2). When comparing the two subgroups of COVID-positive patients, patients with adverse outcomes had a significantly higher CT-SS score (22.6 ± 9.0 vs. 13.8 ± 10.0, *p* < 0.001, Fig. 3). However, when directly comparing the COVID-AO+ subgroup with the Org-PNA control cohort, the CT-SS was significantly lower in COVID-AO+ subgroup (*p* < 0.01), despite a higher RV:LV ratio (Fig. 3, Table 2). This highlights the observation that an increased RV:LV ratio in COVID-AO+ subgroup is out of proportion to the lung parenchymal damage.

### Validation of RV:LV ratio ≥ 1.1 to predict adverse outcomes in COVID-19

To validate the findings of using CTA-based RV:LV ratio ≥ 1.1 to predict adverse outcomes, we reviewed the clinical course of 25 consecutive COVID-19 positive patients who underwent CTA chest between October 1 and October 30, 2020. The baseline characteristics of the patients are summarized in Table 1. In the validation cohort, five patients had an RV:LV ratio ≥ 1.1 (1.2 ± 0.1), and 4 of these had an adverse outcome. The rest of the 20 patients had an RV:LV ratio < 1.1 (0.9 ± 0.1), and of these, only one patient had an adverse outcome. In this validation cohort, the odds of having an adverse outcome were 76:1 with an RV:LV ratio ≥ 1.1.

## Discussion

We report that a dilated RV in the presence of a normal-sized pulmonary artery, quantified on CTA chest imaging in COVID-19 patients, is correlated with adverse outcomes. We identified that RV:LV ratio ≥ 1.1 is a potential marker of adverse outcomes. Moreover, the increased RV:LV ratio in COVID-19 patients was out of proportion to the lung parenchymal changes, quantified with CT-SS. Although this is a retrospective study, our findings speak to the unique pathophysiology of COVID-19 infection.

In the pathophysiology of COVID-19 infection, lung parenchymal damage, hypercoagulable state, direct myocardial damage, and cytokine storm have been implicated [7, 17]. Ciceri et al. have described an endothelial thrombo-inflammatory syndrome in severe COVID-19 infections [29]. This is a result of alveolar viral damage followed by an inflammatory reaction and microvascular thrombosis. Our findings of a dilated RV, out of proportion to the lung parenchymal damage (CT-SS), point to a similar pulmonary vascular injury in severe COVID-19 infections. In comparison with other infections, the COVID-19 has some unique features [30]. However, there is a growing consensus that the pulmonary vascular injury in severe COVID-19 infection is similar to ARDS and should be managed as ARDS [30]. Our findings of the prognostic ability of RV dysfunction and dilation in severe COVID-19 are similar to prior reports of RV dysfunction in ARDS and acute PE [21, 31]. Hence, in addition to biomarkers, the early recognition of RV dysfunction in COVID-19 with imaging could help identify patients who are at risk of adverse outcomes.

Argulian et al. reported an echocardiogram-based study on 105 patients with COVID-19 infection that RV dilation is a marker of poor outcomes [32]. Our findings provide a similar observation that non-invasive imaging methods can provide early recognition of RV strain and may be utilized for appropriate triage and resource utilization. Although an echocardiogram is more readily available, a CTA chest can provide more comprehensive information about clot burden and different lung complications from COVID-19. Overall, our findings suggest that the RV:LV ratio can be consistently quantified on CTA chest, and an RV:LV ratio ≥ 1.1 might predict disease severity in COVID-19 infection.

There are several limitations to our study. A causal relationship between COVID-19 and RV dilation cannot be established due to our study's retrospective nature. Given the variable timeline between CTA chest and COVID testing, we did not report a time-to-event or Kaplan–Meier survival analysis. We suggest that future studies with simultaneous CTA chest and COVID-19 infection test the ability to predict the adverse outcome with a 1.1 cutoff of RV:LV ratio in a larger patient population. The echocardiogram data were limited in our study (*n* = 11).

## Conclusions

In our retrospective study, increased RV:LV ratio ≥ 1.1 on CTA chest in COVID-19 patients is associated with adverse clinical outcomes (ICU admission, endotracheal intubation, or death). RV dilation in severe COVID-19 infections is out of proportion to parenchymal lung damage, which may identify a significant vascular injury pattern similar to ARDS.

## Data Availability

Data are available per request.

## Abbreviations

AO: Aorta
ARDS: Acute respiratory distress syndrome
BMI: Body mass index
BNP: B-type natriuretic peptide
COVID-19: Coronavirus disease 2019
COVID-AO+: COVID-19 patients with adverse outcomes (death, ICU admission, or intubation)
COVID-AO−: COVID-19 patients without adverse outcomes
CTA: Computer tomography angiogram
CT-SS: CT severity score
ECG: Electrocardiogram
ICU: Intensive care unit
LV: Left ventricle
PA: Pulmonary artery
PE: Pulmonary embolism
Org-PNA: Organizing pneumonia
RV: Right ventricle
TTE: Transthoracic echocardiogram
